# Gracilis muscle interposition with primary rectal without urethral repair for moderate sized rectourethral fistula caused by brachytherapy for prostate cancer: a case report

**DOI:** 10.1186/1752-1947-6-323

**Published:** 2012-09-25

**Authors:** Narimantas Evaldas Samalavicius, Raimundas Lunevicius, Rakesh Kumar Gupta, Tomas Poskus, Albertas Ulys

**Affiliations:** 1 Faculty of Medicine, Vilnius University, Oncology Institute, 1 Santariskiu Street, Vilnius, LT 08406, Lithuania; 2 King's College Hospital NHS Foundation Trust, King's Health Partners Academic Health Sciences Centre, Denmark Hill, London, SE5 9RS, UK; 3 Department of Surgery, B.P. Koirala Institute of Health Sciences, Dharan, Nepal; 4 Vilnius University Hospital Santariskiu Klinikos Center Branch, 3 Zygimantu Street, Vilnius, LT 01128, Lithuania

**Keywords:** Brachytherapy, Gracilis interposition, Prostate cancer, Radiotherapy, Rectal repair, Rectourethral fistula

## Abstract

**Introduction:**

There is a 0.16% chance of a rectourethral fistula after prostate brachytherapy monotherapy using Palladium-103 or Iodine-125 implants. We present an unusual case report of a rectourethral fistula following brachyradiotherapy monotherapy for prostate adenocarcinoma. It was also associated with unusual management of the fistula.

**Case presentation:**

A 58-year-old Caucasian man underwent brachyradiotherapy monotherapy as definitive treatment for verified intracapsular prostate adenocarcinoma receiving 56 Iodine-125 implants using a transrectal ultrasound-guided technique. The patient started to complain of severe perineal pain and mild rectal bleeding 15Â months after brachyradiotherapy. A biopsy of mucosa of his anterior rectal wall was performed. A moderate sized rectourethral fistula was confirmed 23Â months after implantation of Iodine-125 seeds. Laparoscopic sigmoidostomy and suprapubic cystostomy were then performed. Long-term cortisone applications in combination with 30 sessions of hyperbaric oxygen therapy, and antibacterial therapies were initiated due to necrotic infection. A gracilis muscle interposition to create a partition between the patient's rectum and urethra in conjunction with primary rectal repair but without urethral repair were performed 6 months later. The 3cm rectal defect was repaired via a 3cm-long horizontal perineal incision. The 1.5cm urethral defect just below the prostate was not repaired. The patient underwent an optic internal urethrotomy 3Â months later for a 1.5cm-long urethral stricture. Several planned preventive urethral buginages were performed to avoid urethral stricture recurrence. At 12Â months postoperatively, there were no signs of a fistula and cancer recurrence. He now has a normal voiding and anal continence.

**Conclusion:**

Severe rectal pain, bleeding, and local anterior necrotic proctitis are predictors of a rectourethral fistula. Urinary and fecal diversion is the first-step operation. Gracilis muscle interposition in conjunction with primary rectal repair but without urethral reconstruction is one of the reconstructive surgery options for moderate 2cm to 3cm rectourethral fistulas. Internal urethrotomy is a procedure for postoperative urethral strictures of 1.5cm in length.

## Introduction

There is a 0.16% chance of a rectourethral fistula (RUF) after prostate brachytherapy (BT) monotherapy using Palladium-103 or Iodine-125 implants [1]. However, if RUFs occur as sequelae of BT, it is more complex comparing with RUF following radical prostate surgery thus creating a more burdensome reconstructive effort [2,3]. Interestingly, theoretical background regarding management of RUFs following BT for prostate carcinoma is limited, yet an online search of the PubMed database using the filter ‘Rectourethral fistula and brachytherapy' revealed only 10 references. We present an unusual case report of a RUF following brachyradiotherapy monotherapy for histologically verified stage I prostate adenocarcinoma that was successfully managed by complex therapy including gracilis muscle interposition surgery with primary rectal but without urethral repair.

## Case presentation

A 58-year-old Caucasian man underwent BT monotherapy as definitive treatment for verified intracapsular prostate adenocarcinoma receiving 56 Iodine-125 implants using a transrectal ultrasound-guided technique. The initial serum prostate-specific antigen level fell from 3.5 to 0.002ng/mL. The patient started to complain of severe perineal pain and mild rectal bleeding 15 months after BT. A biopsy of mucosa of the patient's anterior rectal wall revealed inflammatory changes above the dentate line and cortisone was continuously applied locally. This therapy was stopped 8Â months later when fecaluria, urine leakage per rectum, pelvic pain, orchiepididymitis, and fever were documented. Laparoscopic sigmoidostomy and suprapubic cystostomy were then performed. The patient was referred to a tertiary oncology center because of increasing pain 2Â months after this procedure. An extensive necrotic perifistulous injury was obvious. Thus long-term cortisone applications in combination with 30 sessions of hyperbaric oxygen therapy (HBOT), and antibacterial therapies were initiated. After 6Â months (FigureÂ 1), when clinical resolution of necrotic perifistulous infection was confirmed and distal urinary obstruction was ruled out, a gracilis muscle interposition to create a partition between the patient's rectum and urethra in conjunction with primary rectal but without urethral repair was performed. The 3cm rectal defect was repaired via a 3cm-long horizontal perineal incision with interrupted single layer vicryl sutures. The 1.5cm urethral defect just below the prostate was not repaired. The tendon of the right gracilis muscle was fixed to the left pubic bone. Vicryl was applied to fix the gracilis to the rectal wall and tissues lying around the urethra. Before skin closure, a small suction drain was then placed in the perineal wound. Full healing of the rectal wall was later confirmed 2Â months after reconstructive surgery (FigureÂ 2). Sigmoidostomy take-down operation was performed after 8Â weeks; the patient underwent an optic internal urethrotomy 3Â months later for a 1.5cm-long urethral stricture. The suprapubic cystostomy catheter was removed. A urinary catheter was inserted via the external urethral meatus, and was left in the bladder for 3Â weeks. Several planned preventive urethral buginages were performed to avoid urethral stricture recurrence. At 12 months postoperatively, there were no signs of a fistula or cancer recurrence. He now has a normal voiding and anal continence. 

**Figure 1:**
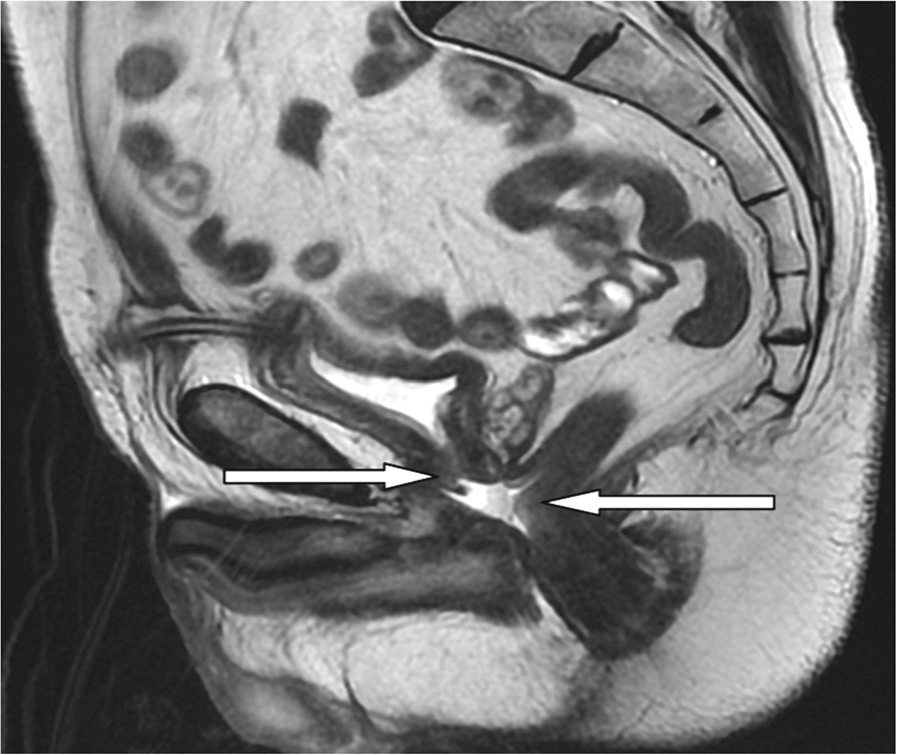
Magnetic resonance image demonstrating moderate sized rectourethral fistula (arrows).

**Figure 2:**
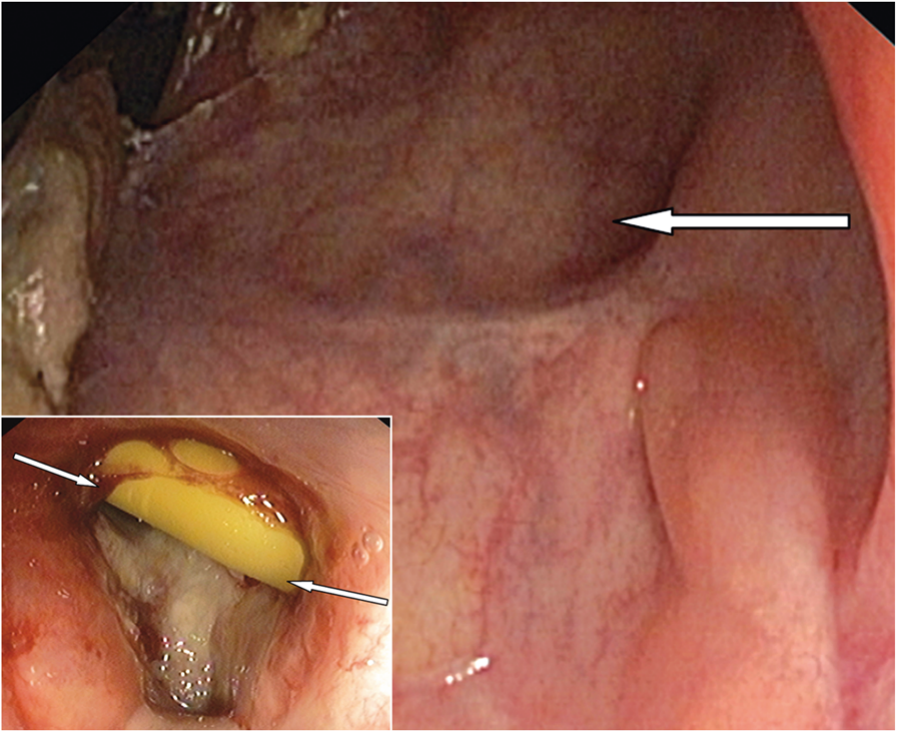
Site of rectourethral fistula (arrow) 2Â months after transverse repair of the rectum and gracilis interposition procedure; at the lower left corner, rectourethral fistula 2Â months before reconstructive surgery (arrows indicate Foley catheter).

## Discussion

In the light of previously described post-radiotherapy RUF size with a mean of 2.8cm [4] and average diameter of 3.2cm [5], the RUF presented in our paper should be described as moderate sized. It is said that patients with a fistula that is greater than 1cm in diameter should undergo gracilis interposition in conjunction with primary rectal repair and buccal mucosa grafting urethral reconstruction [4]. However, the issue of RUF classification and patients' stratification remains open in this clinical setting.

The fistula described is a late sequela of BT. Severe perineal pain, mild bleeding, and local proctitis were predisposing factors. These RUF predictors should be taken into consideration responsibly because a long-term patient's surveillance plan can be established for timely diagnosis of RUF because it can occur a few years after Iodine-125 seed implantation [5]. Furthermore, because an anterior rectal biopsy after prostate radiation has been associated with a higher incidence of RUF formation, an anterior rectal biopsy should not be performed unless there is an obvious cancer staging-related indication for a biopsy. RUF formation after BT is reported to be up to 8.8% if an anterior rectal biopsy is taken after this radiotherapy procedure [1,2].

Long-term local use of cortisone for a conservative treatment of proctitis which is associated with BT is controversial. There are no reports supporting such conservative management of post-BT proctitis. Retrospectively, we assume that long-term steroid therapy for proctitis was a risk factor for RUF. We also postulated that HBOT may result in accelerated healing of injured tissues because it is thought that HBOT improves oxygen supply to wounds and therefore improves their healing. Again, there is no evidence supporting the HBOT in the management of patients with radiation-induced pelvic soft tissue necrosis including proctitis [6].

As there is no standardized treatment for a BT-induced RUF the responsibility for treatment is borne by the physicians in charge. Our chosen surgical strategy resulted in a good outcome and it was similar to an earlier proposed procedure [4]. Nevertheless, the case report highlights one significant difference: there was no attempt to perform substitution urethroplasty either way, by primary repair or buccal mucosa reconstruction [7]. Moreover, postoperative formation of a membranous urethral stricture up to 1.5cm was predicted before and during the reconstructive surgery. Optic internal urethrotomy was a further treatment option because the urethral stricture was 1.5cm in length. Timely gracilis muscle transposition and rectal repair were key elements of successful RUF treatment. Although it is believed that radiotherapy-induced RUFs are much more challenging to reconstruct than inflammatory, iatrogenic, and traumatic RUFs because the magnitude of fibrotic tissue in the area of RUF is extensive [4], it is not the case for every patient. Duration from insertion of radioactive implants to reconstructive surgery is possibly the main factor for the magnitude of fibrotic tissue around the RUF. Finally, in terms of good quality of life, it is believed that transperineal repair with gracilis muscle interposition is an effective treatment for patients with complex RUFs following radiotherapy. However, long-term follow-up studies are needed.

## Conclusion

Prostate adenocarcinoma BT monotherapy may lead to significant RUF-related long-term morbidity. Severe perineal pain, rectal bleeding, and local anterior proctitis are predictors of RUFs. Urinary and fecal diversion is the first-step operation in management of RUFs. Gracilis muscle interposition in conjunction with primary rectal but without urethral reconstruction is one of the reconstructive surgery options for a moderate sized RUF. Internal urethrotomy is a procedure for postoperative urethral stricture 1.5cm in length.

## Consent

Written informed consent was obtained from the patient for publication of this case report and any accompanying images. A copy of the written consent is available for review by the Editor-in-Chief of this journal.

## Competing interests

The authors declare that they have no competing interests.

## Authors' contributions

All authors equally participated in the design of the paper. RKG carried out provisional drafting. TP helped to write a provisional draft. RL performed systematic and final literature search, review, interpretation of literature data, secondary and final drafting. NES carried out the critical revision of the manuscript and participated in presentation of the medical case report. AU acted as a urology consultant. All authors read and approved the final manuscript.
